# Identification of a Putative Novel Genotype of Avian Hepatitis E Virus from Apparently Healthy Chickens in Southwestern Nigeria

**DOI:** 10.3390/v13060954

**Published:** 2021-05-21

**Authors:** Fisayo Temilade Osamudiamen, Olusola Aanuoluwapo Akanbi, Steffen Zander, Daniel Oladimeji Oluwayelu, Claus-Thomas Bock, Patrycja Klink

**Affiliations:** 1 Department of Veterinary Microbiology, University of Ibadan, 200223 Ibadan, Nigeria; ftosamudiamen@gmail.com (F.T.O.); ogloryus@yahoo.com (D.O.O.); 2 Department of Infectious Diseases, Division of Viral Gastroenteritis and Hepatitis Pathogens and Enteroviruses, Robert Koch Institute, 13353 Berlin, Germany; olusola.akanbi@ncdc.gov.ng (O.A.A.); zanders@rki.de (S.Z.); klinkp@rki.de (P.K.); 3 Institute of Tropical Medicine, University of Tuebigen, 72074 Tuebigen, Germany

**Keywords:** hepatitis E virus, avian HEV, layer chickens, novel genotype

## Abstract

Avian hepatitis E virus (aHEV) is the major etiological agent of hepatitis-splenomegaly syndrome (HSS), big liver and spleen disease (BLSD), and hepatic rupture hemorrhage syndrome (HRHS) in chickens. Infections with aHEV cause a significant decrease in egg production and increased mortality in chickens worldwide. However, studies on the prevalence of aHEV in Nigeria are scarce. In this study, serum (*n* = 88) and fecal samples (*n* = 110) obtained from apparently healthy layer chickens from three states in southwestern Nigeria were analyzed by nested reverse transcription-polymerase chain reaction (nRT-PCR) targeting the helicase and capsid gene for the presence of aHEV. Avian HEV was detected in 12.5% (*n* = 11/88) of serum samples and 9.1% (*n* = 10/110) of fecal samples tested. Phylogenetic analysis showed that five of the twelve identified aHEV sequences belonged to genotype 2. The remaining seven sequences were only distantly related to other known aHEV isolates. After amplification of the near-complete ORF2 fragment (1618 bp) and part of the ORF1 (582 bp) of isolate YF40_aHEV_NG phylogenetic analysis revealed a nucleotide sequence identity between 79.0 and 82.6% and 80.1 and 83.5%, respectively, to other known aHEV strains, indicating that the Nigerian isolate YF40_aHEV_NG belongs to a novel aHEV genotype. This is the first report of co-circulation of aHEV genotypes in chickens in Nigeria.

## 1. Introduction

Avian hepatitis E virus (aHEV) is a single-stranded, non-enveloped, positive sense RNA virus belonging to the family *Hepeviridae,* genus *Orthohepevirus* and *Orthohepevirus B* species. The whole genome of aHEV is approximately 6.7 kb in length and consists of a 5′ non-coding region of about 25 bp, followed by three unique open reading frames (ORF1-3), and a 3′ non-coding region with a polyadenylated tail [1,2]. It is considered as a causative agent of hepatitis-splenomegaly syndrome (HSS) and big liver and spleen disease (BLSD) in chickens, turkeys, and wild birds, and the recently reported hepatic rupture hemorrhage syndrome (HRHS) in chickens [3,4,5,6,7]. BLSD and HSS in chickens have been reported since the early 1980s and 1990s from Australia [8] and Canada [9], respectively. However, it was not until the end of the last century, that a genetic relatedness of BLSD and HSS to hepatitis E virus has been shown [10,11,12]. Infection with aHEV may progress from subclinical to having sudden onset of elevated mortality accompanied with drastic reduction in egg production [13]. Chickens affected by HSS and BLSD usually present with enlarged livers and spleens that are obviously above normal, blood-stained ascites, and a prominent keel bone at post-mortem examination. However, it has also been demonstrated that both aHEV RNA and anti-aHEV antibodies can be recovered from healthy chicken flocks [14].

There is only one known serotype of aHEV [2,15] but genomic sequencing has so far revealed four distinct genotypes of aHEV (genotypes 1–4) which are believed to be specifically linked with the host species and geographic origin [16]. Avian HEV strains share approximately 73–100% nucleotide sequence identity with each other but only about 30–50% sequence identity with human and swine HEV [17]. Genotype 1 includes isolates that originated from chickens with BLSD in Australia and Korea [10,18], while genotype 2 has been detected in chickens with BLSD and HSS from the United States, Central Europe, and Spain [12,13,19,20]. It has also been reported that a subtype of avian HEV genotype 2 is structurally comparable to the human and swine HEV [21,22]. Genotype 3 is prevalent in chickens in Europe [23], China, and North America [24], while genotype 4 has been reported from chickens in Hungary and apparently healthy chickens in Taiwan [17,18,21,25,26,27]. Recently, aHEV strains isolated from China and Pakistan have been proposed to belong to novel genotypes [7,28].

Hepatitis E is endemic in several low-income countries in Africa [29]. In Nigeria, HEV is associated with sporadic cases and outbreaks in humans [30,31] and serological evidence has been reported from swine, rabbits, goats, hares, sheep, cattle, and layer chickens [32,33,34,35]. Until recently, when aHEV seroprevalence of 14.6% was reported in commercial layer chickens in Southwestern Nigeria [36], there was no report of the detection of aHEV antibodies among the chicken population in Nigeria. Although Ogbolu and Obi [37] reported the presence of aHEV RNA from chicken fecal samples collected in a study conducted in Lagos and Ogun States, Nigeria, only a small number of samples were tested in that study and no information on the virus genotype was provided. Further published works on HSS in chickens in Nigeria are scanty and the cases were associated with Marek’s disease, avian leukosis and colibacillosis [38,39,40]. In addition, reports of disease outbreaks characterized by reduced egg production in layer chickens, and increased mortality accompanied by lesions of enlarged liver and spleen were also linked to these established diseases with no consideration for aHEV [41]. This study was therefore designed to investigate the occurrence of aHEV in apparently healthy commercial layer chicken flocks in Southwestern Nigeria.

## 2. Materials and Methods

### 2.1. Sample Collection and Processing

Freshly voided, pooled fecal samples (*n* = 110) were gathered from the floor of the pens and blood samples (*n* = 88) were obtained from apparently healthy layer chickens aged 24–52 weeks from 36 poultry farms located in Ogun, Osun and Oyo States, Southwestern Nigeria between October 2018 and October 2019. On all farms, chickens were reared on battery cage management system. Some of the farms had a previous history of reduced egg production and mortalities. However, all sampled chickens were asymptomatic. The study protocol was reviewed and approved by the Animal Care and Use Research Ethics Committee (ACUREC), University of Ibadan, Ibadan, Nigeria (UI-ACUREC/17/0042).

To prepare a 10% (*w*/*v*) suspension of each sample, 100 mg of feces was vigorously mixed with 1 mL of PBS; the suspension was then centrifuged at 13,000 rpm (4 °C) for 5 min. Clarified supernatants were transferred into labeled micro tubes which were screw capped and stored at −80 °C. To obtain serum, the blood samples (2 mL each) were collected by jugular venipuncture, allowed to clot, and kept at 4 °C overnight. Thereafter, they were centrifuged at 8000 rpm for 1 min. All serum samples were then kept frozen at −80 °C until November 2019 when they were thawed and aliquots spotted on Whatman^®^ 903 protein saver cards (Cytiva, formerly GE Healthcare Life Sciences, Marlborough, USA). Thirty µL of the clarified fecal supernatant and serum samples were slowly applied to one dotted circle of Whatman^®^ 903 protein saver cards. For each sample, five spots were made. The filter cards were allowed to air-dry and were subsequently placed in labeled zip-lock bags together with two desiccant bags and closed tightly. All the plastic bags were then stored at −20 °C until shipment at room temperature to the Robert Koch Institute, Berlin, Germany.

### 2.2. Viral RNA Extraction

Viral RNA extraction from dried fecal and serum spots was performed using the QIAamp^®^ viral RNA kit (Qiagen, Hilden, Germany). Two dried fecal and serum spots each were punched out from the Whatman^®^ cards and put into a 1.5 mL reaction tube. After adding 560 µl of buffer AVL/carrier RNA-mix, the tubes were shaken for 1 h at room temperature using an Eppendorf^®^ thermo mixer 5433. The eluate (~450 µl) was transferred into a new labeled reaction tube and subsequent RNA extraction was performed, following the manufacturer’s instructions with elution in 60 µl of elution buffer. The concentration of nucleic acids in each sample was measured using a Nanodrop^®^ device and thereafter stored at −80 °C until analyzed.

### 2.3. Molecular Detection and Characterization of aHEV RNA in Fecal and Serum Samples

For the detection of aHEV-RNA, two degenerate primer pairs targeting the helicase and capsid regions were used as described previously [14]. Further characterization of the viral RNA was performed using overlapping in-house primers. Primers were designed with Geneious Prime software version 2020.0.5 (Biomatters, New Zealand) based on an alignment with aHEV full length sequences available at GenBank (*n* = 14) (Table 1).

For the first strand complementary DNA (cDNA) synthesis, the Maxima H minus Reverse Transcriptase (Thermo Scientific^®^, Vilnius, Lithuania) was used according to manufacturer’s instructions. Two-step RT-PCR amplification was performed with the Qiagen HotStarTaq master mix kit (Qiagen, Hilden, Germany) using a Biometra Trio thermo cycler (Biometra, Jena, Germany). The PCR conditions for the overlapping PCR assays were as follows: For the first round PCR, the PCR mixture in a 25 μL final volume comprised of 2 μL 2X Qiagen HotStarTaq master mix, 0.5 µl dNTPs (10 mM), 0.625 μL each of 10 µM forward and reverse primers, 1 μL cDNA template, and 20.25 μL RNase/DNase-free water to make up the volume. The following cycling conditions were used for the first reaction PCR: initial incubation at 94 °C for 4 min, followed by 40 cycles of 94ºC for 10 s, 55ºC (Ramp 1 ºC/s) for 30 sec, and 72 °C for 60 s, and a final incubation step at 72 °C for 2 min. For the second PCR, the PCR mixture in a 25 μL final volume comprised of 2 μL 2X Qiagen HotStarTaq master mix, 0.5 µl dNTPs (10 mM), 0.625 μL each of 10 µM forward and reverse primers, 1 μL first PCR template, and 20.25 μL RNase/DNase-free water to make up the volume. The PCR cycling condition were initial incubation at 95 °C for 15 min, followed by 30 cycles of 94 °C for 10 s, 55 °C (Ramp 1 °C/s) for 60 s, and 72ºC for 25 s, and a final incubation step at 72 °C for 2 min. A sample was considered positive when detected by the second PCR. An infectious clone of pT7-aHEV plasmid (kindly provided by Professor X. J. Meng, Virginia Polytechnic Institute and State University, Blacksburg, USA) was used as positive control for the nested PCR. The PCR products were visualized on a 1.5% agarose gel stained with Gel Red^®^ (Biotium^®^, Northern California, USA) using a BioDocAnalyze (Analytik Jena^®^, Jena, Germany). The sizes of the DNA fragments were estimated by comparison with a 100 bp DNA ladder (Thermo Fisher Scientific^®^, Berlin, Germany).

### 2.4. Sequencing and Phylogenetic Analyses

PCR products with bands at the expected sizes were enzymatically cleaned up using the ExoSAP-IT™ Express PCR Product Clean Up kit (Thermo Fisher Scientific^®^, Berlin, Germany). Sanger sequencing was performed directly on both strands at the sequencing facility of the Robert Koch Institute, Berlin, Germany, using 0.5 µl Big Dye version 3.1 (Life Technologies, Applied Biosystems, Darmstadt, Germany). The resulting aHEV sequences were compared with reference aHEV strains available at GenBank. Multiple sequence alignments with reference strains were performed using the MAFFT method with default options in Geneious Prime software version 2020.0.5 (Biomatters, New Zealand). Phylogenetic trees were constructed by maximum likelihood method with 1000 standard non-parametric bootstrap replicates using IQ-TREE 1.6.12 [42]. The best fitting model was determined using the substitution model test included in IQ-TREE [43]. Trees were graphically adjusted using iTOL [44].

### 2.5. Statistical analysis

The statistical analysis was performed using the Fisher’s exact 2-tailed test with 95% confidence interval in Stata (STATA^®^ statistical software Version 14).

## 3. Results

### 3.1. Molecular Detection of aHEV

Fecal (*n* = 110) and serum samples (*n* = 88) were collected from apparently healthy commercial layer chickens aged 24 to 52 weeks, reared on battery cage management system from Ogun, Osun, and Oyo States in southwestern Nigeria. Two nested RT-PCR assays targeting partial sequences of the helicase (ORF1) and capsid (ORF2) genes, respectively, revealed the presence of specific aHEV RNA in 12.5% (*n* = 11/88) of serum and 9.1% (*n* = 10/110) of fecal samples tested (Table 2). No statistically significant difference in prevalence were observed when ages of chickens and locations was observed were compared.

### 3.2. Phylogenetic Analysis and Genotyping

Direct sequencing of the 21 aHEV-positive PCR products (*n* = 21/198) obtained for helicase and capsid genes resulted in a total of 12 sequence samples (3 helicase gene sequences and 9 capsid gene sequences), respectively, that were of sufficient quality to allow for phylogenetic analysis. The three helicase gene sequences belonged to two serum samples collected from a 25- and a 46-week-old chicken in Osun State and one fecal sample collected from a 32-week-old chicken in Oyo State (Table 3). The nine capsid gene sequences were obtained from samples from all three states: 3 fecal (45–50-week-old chickens) and 1 serum sample (24-week-old chicken) from Ogun State, 1 fecal sample (32-week-old chicken) from Osun State and 3 fecal (38–40-week-old chickens) and 1 serum sample (50-week-old chicken) from Oyo State, respectively. There was no correlation between nucleotide sequence and type of sample collected (whether serum or fecal sample). Further, all the isolates in this study originated from different farms and states except two isolates, YF40-aHEV-NG and YF41-aHEV-NG, that were from one farm with chickens of the same age and the same sampling date.

**Figure 1:**
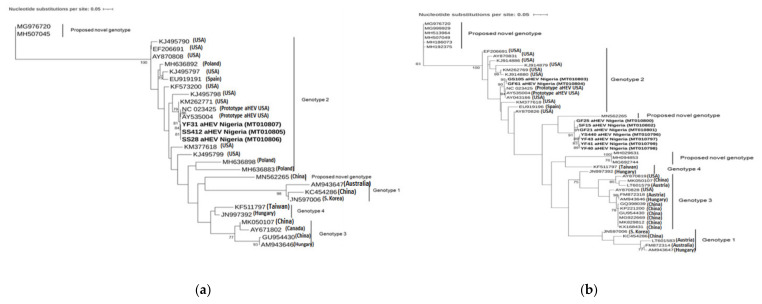
(**a**) Phylogenetic tree based on the 302 bp long nucleotide sequences of the partial helicase gene (ORF1) and (**b**) 194 bp long nucleotide sequence of the partial capsid gene fragment (ORF2) of the Nigerian isolates and other known aHEV strains. The evolutionary history was inferred by maximum likelihood method with the TIM3 +F + 1 + G4 and Tne + G4 model using IQTREE 1.6.12. Bootstrap values >75% are shown. The Nigerian aHEV isolates are in bold.

The 12 sequences of the Nigerian aHEV strains obtained from this study were compared with complete and near-complete genomic sequences of aHEV available in GenBank including sequences of proposed novel genotypes of aHEV. Maximum likelihood trees were constructed for the partial helicase gene sequences (302 bp) and the partial capsid gene sequences (194 bp). Based on the result of the model tester implemented in IQ-TREE for the partial helicase gene sequences, the TIM3 + F + I + G4 model was used (Figure 1a), while for the partial capsid gene sequences, the TNe + G4 model fitted best (Figure 1b). All three Nigerian aHEV helicase gene sequences (*n* = 3) from this study clustered together with the prototype aHEV NC023425 in the genotype 2 sharing between 92.2% and 92.4% identity.

The Nigerian aHEV capsid gene sequences (*n* = 9) formed two separate clusters within the phylogenetic tree. The first cluster contained two sequences and was related to the prototype aHEV NC023425 in genotype 2 (81.4–83.3% nucleotide identity). The remaining seven sequences built one cluster (95.4%–100% nucleotide sequence identity) which showed a nucleotide sequence identity to all other known aHEV strains between 74.4% and 84.2% with the highest sequence identity (82.8–84.2%) to a potential novel genotype (MN652265), isolated from a Silkie fowl in China [28].

**Figure 2:**
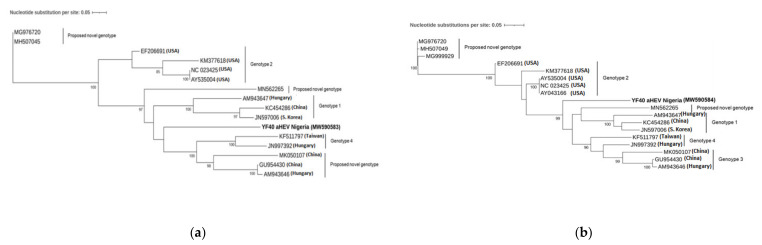
(**a**) Phylogenetic tree based on the 564 bp long nucleotide sequences of the partial PCP/HVR gene (ORF1) and (**b**) 1618 bp long nucleotide sequence of the partial capsid gene fragment (ORF2) of YF40_aHEV_NG and other available aHEV strains. The evolutionary history was inferred by maximum likelihood method with the TIM3 +F + 1 + G4 and TIM2 + F + G4 model using IQTREE 1.6.12. Bootstrap values >75% are shown. The Nigerian aHEV isolates are in bold.

In order to further characterize the sequences clustering together with the potential novel genotype (MN652265), primers for the amplification of the full-length genome were designed. Subsequent analysis was performed with one sample (YF40_aHEV_NG) as a representative of the seven sequences clustering with the putative novel aHEV genotype. Phylogenetic analysis of the ORF1 fragment (564 bp; TIM2 + F + I + G4 model) showed that the isolate is only distinctly related to all other aHEV strains with a nucleotide sequence identity between 69.2% and 74.4% (Figure 2a). As the ORF1 fragment includes the highly divergent hypervariable region (HVR), phylogenetic analysis was additionally performed excluding this region (372 bp; TNe + I + G4 model) resulting in the same distinct relationship to all other aHEV isolates, however with a nucleotide sequence identity between 79.0 and 82.6% (tree not shown). On the amino acid level, the sequence of the partial ORF1 Nigerian isolate showed an identity between 61.9 and 81.0% and 80.0 and 95.4% (including or excluding the HVR region, respectively) to other known aHEV sequences. Phylogenetic analysis of the ORF2 fragment (1618 bp; TIM2 + F + G4 model) showed that the isolate is only distantly related to all other known aHEV strains with a nucleotide sequence identity between 80.1 and 83.5% (Figure 2b). The partial ORF2 sequence showed an amino acid identity between 90.2 and 98.3%) to other known aHEV sequences.

## 4. Discussion

Avian HEV infections have been reported in several countries in North America, Europe, Australia, and Asia from both apparently healthy as well as HSS and BLSD infected poultry [8,12,19,26]. In Nigeria, cases of human HEV outbreaks have been reported [30,31]. Further, serological evidence of HEV is known from swine, rabbits, goats, sheep, cattle [32,34,35], and a 14.6% seroprevalence of aHEV was recently reported in layer chickens in Nigeria [36]. Only one study conducted in Ogun and Lagos Statesfound aHEV RNA in chicken feces samples. However, only a limited number of samples were analyzed and the genotype information from the analysis was not available [37]. So far, HSS in Nigeria has been associated with other diseases, e.g., Marek’s disease, avian leukosis, and colibacillosis but the awareness of aHEV is lacking [38,39,40,41].

According to Billam et al. [45], the presence of aHEV can only be detected by serological or molecular methods since the virus does not replicate in any known cell culture systems and virus isolation can only be done using SPF chickens. The RT-PCR technique is, therefore, the preferred method of identifying aHEV. In the present study, a nested RT-PCR, previously described by Sun and colleagues [14] was used to detect partial helicase and capsid gene fragments of aHEV in fecal and serum samples of apparently healthy commercial layer chickens in southwest Nigeria. The detection rate of aHEV in fecal (9.1%) and sera (12.5%) samples of layer chickens in this present study is higher than the prevalence of aHEV RNA in feces (0%) and sera (1.2%) reported from laying hens in Spain. The higher positivity rate recorded in sera than in feces could be due to the possibility of low virus shedding in feces at the time of sample collection [19]. Sun et al. [14] reported that chickens infected naturally with aHEV generally seroconvert between 15 and 17 weeks. Therefore, our results suggest that virus shedding and viraemia in the chickens might have cleared prior to the sample collection time because all the chickens sampled in this study were older than 23 weeks of age. In contrast, the 9.1% detection rate of aHEV RNA in fecal samples in this study is substantially lower than previously reported from older layers and broiler breeders (38–60-week-old) in South Korea (23%), in 17-week-old pullets, and 46-week-old hens (62.9%) in two different farms with HSS outbreaks in the United States of America [18,46]. Our findings suggest a natural, subclinical infection of commercial layer chicken flocks with aHEV in Ogun, Osun, and Oyo states, southwest Nigeria, since during the time of sampling the chickens showed no obvious signs of disease and, in each state, at least one sample was positive for aHEV RNA.

Phylogenetic analysis of the sequences obtained in this study showed the presence of two different genotypes of aHEV in the sampled chickens: genotype 2 aHEV and a putative novel genotype of aHEV. Five of the twelve isolates were closely related to each other but distinctly clustered with the prototype aHEV (AY535004). This finding is consistent with the report of Sun et al. [14] that avirulent strains of aHEV isolated from healthy chickens do cluster together and are genetically related but nevertheless distinct from prototype aHEV and other isolates obtained from chickens with HSS (virulent strains).

The majority of the sequences from Nigerian aHEV strains identified in the present study (n = 7/12) formed a cluster with 95.4–100% nucleotide sequence identity to each other but showed only a low nucleotide sequence identity to other aHEV isolates (ORF2 74.4–84.2%). However, as the sequences were too short for a reliable phylogenetic analysis, (194 bp) further PCR assays were designed and performed resulting in two longer fragments of the Nigerian isolate YF40_aHEV_NG (ORF1 (PCP/HVR) 564 bp and near-complete ORF2 (capsid) 1658 bp). The phylogenetic analysis of these longer sequences still revealed only a distant relationship to all other known aHEV genotypes (ORF1 excluding HVR region: 79.0–82.6% and ORF2: 80.1–83.5%). The new isolate, YF40_aHEV_NG, is located in a single branch distinctly separated from all other reference strains, both in the ORF1 region and the ORF2 region, indicating a proposed novel aHEV.

In this study, two different genotypes of aHEV have been identified to co-circulate among commercial layer flocks in Nigeria indicating a widespread distribution of different aHEV genotypes in southwestern Nigeria. In addition, this finding corroborates reports of heterogeneity of aHEV and the fact that there is no limit to geographic distribution of the virus, even though certain genotypes maybe more prevalent in some locations than others [14,18]. While cross-species transmission of aHEV to turkeys has been demonstrated [47], experimental infection of non-human primates to evaluate the zoonotic potential of the virus was unsuccessful [1]. Further, the genomic organization of the HEV genome in human and animal strains is similar. However, genomic sequence analyses of aHEV isolates have shown a nucleotide identity of only 50–60% with human and swine HEVs [48]. Therefore, the detection of genotype 2 and the putative novel genotype aHEV in apparently healthy layer chickens in this study may not be of any immediate public health consequence but rather generate questions as to whether HSS occurs more often in Nigeria but is mostly undiagnosed or misdiagnosed as fatty liver syndrome. However, infections with aHEV are associated with increased mortality and decreased egg production leading to economic losses in the poultry industry. Further studies including different geographic locations are needed to assess the prevalence and distribution of aHEV in Nigeria. Molecular analysis of the circulating strains will help to understand the dynamics of aHEV infection in Nigeria and will enable the implementation of targeted measures to prevent infection or spread of the disease in affected flocks.

Full-length sequence of the virus is necessary for the assessment of a novel genotype but probably due to the fragmented or degraded RNA on the dried spots and the shortage of the sample materials, it was not possible to amplify the complete genome of the Nigerian aHEV isolate, YF40_aHEV_NG. This is one limitation of the study, as the fresh, original samples were frozen before and after spotting (dried serum spots) and after shipment at room temperature to the RKI. Freeze-thawing of samples can easily lead to fragmentation and degradation of RNA. Nevertheless, whole genome based phylogenetic analysis is necessary to characterize the novel isolate and further assess its genetic relationship to other aHEV isolates. Moreover, infectivity assays would be necessary to ascertain the pathogenicity of the new strain. However, as all the chickens tested in this study were apparently healthy and egg production was not reduced, the strains seem to have a rather low pathogenicity. A further limitation of the study is that the sensitivity of the PCR assays used could not be ascertained so that false-negative samples due to a low viral load cannot be excluded. Further, studies indicate that the virus concentration might be lower in dried serum spots compared to serum samples. Although studies on the stability and viral load of HEV on filter spots have not been performed until now, both, the degradation of RNA as well as the potentially lower virus concentration on the spots might have led to false-negative results [49,50].

## 5. Conclusions

In summary, we identified and characterized aHEV strains in apparently healthy commercial layer chickens in southwestern Nigeria and showed that genotype 2 and a genotype, which is only distantly related to all other known genotypes, co-circulate in the study area. We therefore propose a novel aHEV genotype; however, more sequences are needed to officially assess a new genotype. Since information on the epidemiological status of aHEV is currently lacking in Nigeria, we advise that the virus be considered as an important disease agent that requires continuous surveillance in chicken farms. In addition, it should be included as a differential diagnosis in cases that present with hepatitis-splenomegaly, reduced egg production, and sudden onset of morbidity with high mortality in layer chickens in Nigeria.

## Figures and Tables

**Table 1:** Primers for the characterization of aHEV.

Primer	Location *	Size (bp)	Sequence (5′-3′)
**ORF1**			
aHEV-8f	1302–1320	585	TCGTCAGCTCGCCACATGG
aHEV-13r	1869–1886		AAAACAGCAAGGACCTCC^a^
aHEV-9fn	1303–1320	583	CGTCAGCTCGCCACATGG
aHEV15rn	1867–1885		AAACAGCAAGGACCTCCTC
**ORF2/3**			
aHEV-3f	4807–4825	739	CGTGACAACTCAGCCCAGTG^b^
aHEV-25r	5523–5545		GGTCAGCTTTGATGGTGTGTGG
aHEV-7fn	4809–4829	732	GACAACTCAGCACAGTGGAGC^c^
aHEV-26rn	5520–5540		AGCTTTGATGGTGTGTGGTGC
**ORF2 (Part 1)**			
aHEV-27f	5429–5448	370	ACTCGGCATGGTTGATCTGG
aHEV-28r	5777–5798		AGCATCCTCAACCGACATGTAC
aHEV-29fn	5473–5492	289	TATCACCCGGCAACACGAAC
a-HEV30rn	5742–5761		TCACCGTTCGCATTCCCAAC
**ORF2 (Part 2)**			
aHEV-24f	5684–5702	796	TGGCYTGCCCTCGACATTG
aHEV-2r0	6459–6479		AGCTTTGATGGTGTGTGGTGC
aHEV-23fn	5689–5707	778	TGCCCTCGACATTGTTGCG
aHEV-21rn	6448–6466		TGGTCTTCAGGTGCCTGGC

f: forward; r: reverse; n: nested; Sequence column: a: [24]; b: [15]; c: [7]; * based on prototype aHEV NC023425.

**Table 2:** Occurrence of aHEV in fecal and serum samples collected from three states in Nigeria.

States	Fecal Samples			Serum Samples		
	Age (w) (Median ± IQR)	No. Tested	No. Positive (%)	Age (w) (Median ± IQR)	No. Tested	No. Positive (%)
**Ogun**	30–52 (45 ± 12)	32	3 (9.4)	24–52 (42 ± 26)	20	2(10.0)
**Osun**	32–52 (42 ± 11)	31	2(6.5)	25–52 (42 ± 17)	29	5(17.2)
**Oyo**	32–48 (45 ± 8)	47	5(10.6)	24–52 (38 ± 22)	39	4(10.3)
**Total**		**110**	**10(9.1)**		**88**	**11(12.5)**

No.: number; w: weeks; IQR: interquartile range.

**Table 3:** Characteristics of the sequenced isolates from this study.

ID	GenBank Number	State	Age (Weeks)	Gene	Type	Genotype
GF25	MT010800	Ogun	45	Capsid	Fecal	Putative novel genotype
GF21	MT010801	Ogun	45	Capsid	Fecal	Putative novel genotype
GF 61	MT010804	Ogun	50	Capsid	Fecal	Genotype 2
GS105	MT010803	Ogun	24	Capsid	Serum	Genotype 2
SF15	MT010802	Osun	32	Capsid	Fecal	Putative novel genotype
SS28	MT010806	Osun	25	Helicase	Serum	Genotype 2
SS412	MT010805	Osun	46	Helicase	Serum	Genotype 2
YF31	MT010807	Oyo	32	Helicase	Fecal	Genotype 2
YF40	MT010798	Oyo	38	Capsid	Fecal	Putative novel genotype
	MW590583			PCP/HVR		
	MW590584			Capsid		
YF 41	MT010799	Oyo	38	Capsid	Fecal	Putative novel genotype
YF43	MT010797	Oyo	40	Capsid	Fecal	Putative novel genotype
YS440	MT010796	Oyo	50	Capsid	Serum	Putative novel genotype

## Data Availability

The gene sequences obtained in this study have been submitted to the GenBank Nucleotide Sequence Database under the accession numbers MT010796—MT010807 and MW590583—MW590584. All available data are presented in this manuscript.
